# Supplementary material for: Temperature-Related Short-Term Succession Events of Bacterial Phylotypes in Potter Cove, Antarctica
Source: Genes (Basel). 2023 May 8;14(5):1051. doi: 10.3390/genes14051051 (PMC10218438; doi:10.3390/genes14051051)
Supplement: Supplementary file 1 [file genes-14-01051-s001.zip › genes-2346336-supplementary.pdf]

Supplementary Material 1

1. DNA extraction protocol

Zirconium and glass beads of various diameter (0.1, 0.7 and 1 mm) and 0.6 mL CTAB (cetyltrimethyl-ammonium bromide) buffer were added to ~ 0.5 g of the sample. Then 60 µl of 10% sodium dodecyl sulfate (SDS), 60 µl of 10% N-Lauroylsarcosin and 20,8 µl Proteinase K (10 mg/ml) were added. Samples were incubated for 1 h at 60 °C. After incubation 0,5 ml Phenol-Chloroform-Isoamylalkohol (25:24:1) mixture was added in each tube. Samples were then homogenized on a vortexer for 10 min at highest speed and then centrifuged at 16000×g for 10 min at 4 °C. The aqueous phase was transferred into new reaction tubes, washed with 0,6 mL of chloroform-isoamylalcohol (24:1) and centrifuged at 16000× g for 10 min at 4 °C. Again, the aqueous phase was transferred into new reaction tubes and mixed with 1200 µl of 30% polyethylene glycol (w:v) in 1.6 M NaCl and 2 µl of LPA was added. After 1 h incubation at room temperature, samples were centrifuged at 17000× g for 60 min at 4 °C. The supernatant was removed and the pellet was washed with 800 µl of ice-cold 70% ethanol. After centrifugation at 17000×g for 10 min the supernatant was removed and the nucleic acid pellet was air-dried and finally dissolved in 50 µL ultra-pure water.

Genes **2023**, *14*, 1051. https://doi.org/10.3390/genes14051051 www.mdpi.com/journal/genes
